# 81007 Training Biomedical Engineers in Regulatory Science: Critical Role of Experts from Industry and FDA

**DOI:** 10.1017/cts.2021.674

**Published:** 2021-03-30

**Authors:** Aaron E. Lottes, Andrew O. Brightman

**Affiliations:** Purdue University Weldon School of Biomedical Engineering

## Abstract

ABSTRACT IMPACT: Lack of regulatory knowledge and education is a key barrier to the translation of medical devices and we describe the design and results for a university graduate-level course providing training on medical device regulatory submissions for approval that can help fill this unmet need and improve and accelerate translational success. OBJECTIVES/GOALS: Within the Indiana CTSI, the Medical Technology Advance Program (MTAP) in the Purdue University Weldon School of Biomedical Engineering (BME) offers three courses in regulatory science and regulatory affairs for medical devices. One course is focused on regulatory submissions for approval, and this report details the course design and evaluation. METHODS/STUDY POPULATION: For Fall 2020, the Regulatory Submissions for Approval course was enhanced to increase participation from regulatory professionals in US FDA and industry, with the core content, curriculum and course design led by BME faculty. The course was taught two days per week and included both in-person and remote (synchronous or asynchronous) attendance options. During the first class session each week a topic was covered in standard lecture format by BME faculty with industry regulatory experience. During the second class session, guests from both industry and FDA were invited to provide in-depth discussion on the topic, share perspectives and viewpoints, present real-world examples, experiences, and case studies, and answer student questions. An end of semester survey evaluated the effectiveness of the course design. RESULTS/ANTICIPATED RESULTS: Medical Device regulatory submissions and related activities were taught including product classification, presubmissions and meetings, 510(k), de novo, EUA, PMA, HDE, and advisory panels. FDA history, regulatory careers, regulatory science, and EU, China, and Japan regulations were also discussed. Overall, 29 speakers from FDA and industry participated live via video calls. A survey completed by 21/23 studentsrevealed overall satisfaction: all reported increased regulatory understanding and 20/21 learned ‘a lot’ or ‘an incredible amount’. The weekly lecture was the top factor contributing to learning, and guest speakers were the next most important factors. Nearly all students indicated FDA and industry speakers were ‘very’ or ‘extremely’ valuable/helpful. Additional results will be presented. DISCUSSION/SIGNIFICANCE OF FINDINGS: The three courses are designed to improve medical device translation by training students to better understand regulatory processes and pathways. Survey results and feedback indicated this course was successful. Continued participation from FDA and industry is critical to the learning. Additional case studies will also help enhance learning.

